# Gene Therapy Vector Encoding Neuropeptide Y and Its Receptor Y2 for Future Treatment of Epilepsy: Preclinical Data in Rats

**DOI:** 10.3389/fnmol.2020.603409

**Published:** 2020-12-04

**Authors:** Julia Alicja Szczygieł, Kira Iben Danielsen, Esbjörn Melin, Søren Hofman Rosenkranz, Stanislava Pankratova, Annika Ericsson, Karin Agerman, Merab Kokaia, David Paul Drucker Woldbye

**Affiliations:** ^1^Department of Neuroscience, University of Copenhagen, Copenhagen, Denmark; ^2^Experimental Epilepsy Group, Epilepsy Centre, Lund University Hospital, Lund, Sweden; ^3^CombiGene AB, Medicon Village, Lund, Sweden

**Keywords:** NPY, Y2, learning and memory, AAV viral vector, hippocampus, gene therapy

## Abstract

Gene therapy to treat pharmacoresistant temporal lobe epilepsy in humans is now being developed using an AAV vector (CG01) that encodes the combination of neuropeptide Y and its antiepileptic receptor Y2. With this in mind, the present study aimed to provide important preclinical data on the effects of CG01 on the duration of transgene expression, cellular tropism, and potential side effects on body weight and cognitive function. The CG01 vector was administered unilaterally into the dorsal and ventral hippocampus of adult male rats and expression of both transgenes was found to remain elevated without a sign of decline at 6 months post-injection. CG01 appeared to mediate expression selectively in hippocampal neurons, without expression in astrocytes or oligodendrocytes. No effects were seen on body weight as well as on short- or long-term memory as revealed by testing in the Y-maze or Morris water maze tests. Thus these data show that unilateral CG01 vector treatment as future gene therapy in pharmacoresistant temporal lobe epilepsy patients should result in stable and long-term expression predominantly in neurons and be well tolerated without side effects on body weight and cognitive function.

## Introduction

Epilepsy is the fourth most common disorder of the central nervous system, affecting up to 1% of the world population (Fiest et al., 2017). Since up to 1/3 of epilepsy patients remain resistant to currently available anti-epileptic therapeutics (Picot et al., 2008; Brodie et al., 2012), there is a great unmet need to explore novel treatment avenues. In recent years, gene therapy with viral vectors has emerged as an attractive alternative treatment strategy, particularly for focal epilepsies that also account for the greatest proportion of epilepsies (Wykes and Lignani, 2018). Several different targets have been suggested for therapeutic gene regulation, including neuropeptides [e.g., neuropeptide Y (NPY), galanin, dynorphin], potassium ion channels, and designer receptors exclusively activated by designer drugs (DREADDs; Wykes et al., 2012; Simonato, 2014; Agostinho et al., 2019; Weston et al., 2019). The most frequent type of pharmacoresistant epilepsy is mesial temporal lobe epilepsy (mTLE) with hippocampal sclerosis (Blümcke et al., 2012). Many studies have established the seizure-suppressant effects of NPY against seizures in the hippocampus both in rodents (Woldbye et al., 1996, 1997, 2005; Vezzani et al., 1999; Klemp and Woldbye, 2001) and hippocampal slices from pharmacoresistant human epilepsy patients (Patrylo et al., 1999; Ledri et al., 2015; Wickham et al., 2019). NPY elicits its biological actions in the brain mainly by binding to Y1, Y2, and Y5 receptors, members of a G-protein coupled receptor superfamily (Berglund et al., 2003). In the hippocampus, the seizure-suppressant effects of NPY appear to be mediated primarily *via* activation of Y2 receptors (El Bahh et al., 2005) while Y5 receptors may also play a role particularly outside the hippocampus (Woldbye et al., 1997, 2005; Marsh et al., 1999). In contrast, Y1 receptors appear to act in an opposite manner (Benmaamar et al., 2003; Lin et al., 2006; Olesen et al., 2012).

Using adeno-associated viral (AAV) vectors, it has been shown that hippocampal overexpression of NPY (Richichi et al., 2004; Noè et al., 2008; Noe et al., 2010; Gøtzsche et al., 2012) and/or its antiepileptic receptor Y2 (Woldbye et al., 2010; Ledri et al., 2016) has antiepileptic effects *in vivo* in rodents. A similar seizure-suppressant effect has been reported after AAV-mediated overexpression of NPY or the Y2 agonist NPY13–36 in the piriform cortex (Foti et al., 2007). Combined overexpression of NPY and Y2 in the hippocampus exerted a superior seizure-suppressant effect compared to single transgene expression (Woldbye et al., 2010). To test NPY/Y2 combination gene therapy for human patients with mTLE, we recently provided proof-of-concept with a single vector (CG01) that mediates simultaneous hippocampal overexpression of NPY and Y2 using a translational chronic epilepsy model (Melin et al., 2019). In this model, AAV injection was applied successfully into the hippocampal seizure focus *after* spontaneous recurrent seizures were established. In the present study, we further conducted a series of preclinical experiments in rats to provide important knowledge of the expression and potential side effects of CG01 before future clinical testing, including duration of transgene expression, cells types targeted (i.e., cellular tropism), as well as effects on body weight and cognitive function.

## Materials and Methods

### Animals

All procedures were performed following the Danish Animal Experiments Inspectorate and approved by the local Ethical Committee for Laboratory Animal Research. A total of 52 adult male Wistar rats (Charles River; 200–220 g on arrival) were housed in standard plastic cages on a 12 h light/dark cycle with *ad libitum* access to food and water and adapted for 7 days before experiments.

### Viral Vectors

Two recombinant serotype-1 AAV vectors kindly provided by CombiGene AB (Lund, Sweden) were used in the study: CG01 which encodes human pre-pro-neuropeptide Y (NPY) and its receptor Y2 (AAV1-CAG promotor-pre-proNPY-IRES-hY2-WPRE-BGHpA) and CG07 which is an empty control vector (AAV1-CAG promotor-EMPTY-WPRE-BGHpA; Melin et al., 2019). Both vectors were driven by a synthetic CAG promoter (chicken beta-actin promoter hybridized with the CMV immediate early enhancer sequence). An internal ribosome entry site (IRES) located between the two transgenes assured translation of both.

### AAV Vector Surgery

**Figure 1:**
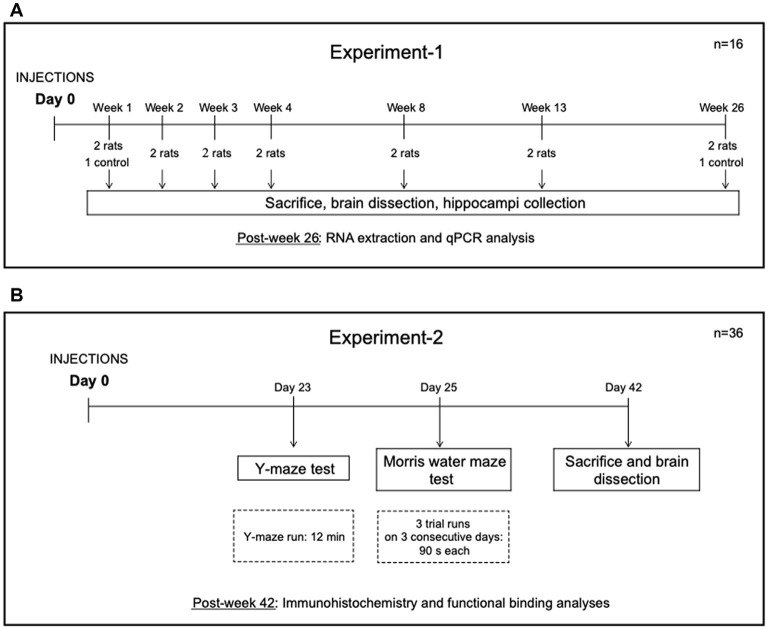
Design of Experiment-1 **(A)** and Experiment-2 **(B)**.

Two separate experiments were performed. The experimental design is shown in Figure 1. In Experiment-1, 14 rats were injected unilaterally in the hippocampus with CG01 vector and subsequently sacrificed at 1, 2, 3, 4, 8, 13, and 26 weeks post-injection (*n* = 2). Also, two treatment-naïve rats were sacrificed at 1 and 26 weeks, respectively. In Experiment-2, 36 rats were randomly allocated to three groups (*n* = 12 per group). The rats in the first group were left untreated (naïve), the second and third groups of rats were injected unilaterally in the hippocampus with CG07 and CG01 vectors, respectively. Animals were subjected to two behavior tests starting 3 weeks after the vector administration.

Before the intracerebral injection, rats were weighed and anesthetized using a 4% isoflurane-oxygen mixture. Anesthesia was maintained with a 1–2.5% isoflurane-oxygen mixture. Each rat received 1 μl/g s.c. injection of temgesic (0.3 mg/ml) and, in the scalp region, 1 μl/g s.c. injection of a mixture of lidocaine (10 mg/ml) and mepivacaine (10 mg/ml). Rats were injected in the right hippocampus in two areas using the following coordinates: dorsal hippocampus (AP −3.3 mm, ML +1.8 mm, DV −2.6 mm relative to dura); ventral hippocampus (AP −4.8 mm, ML +5.2 mm, DV −6.4 mm and −3.8 mm relative to dura) using a stereotaxic frame (Kopf, Sweden). Injections were performed with a 5-μl Hamilton syringe mounted with a glass pipette. The vector of choice was injected at each site in a volume of 1 μl with a speed of 0.1 μl/min and a final concentration of 1 × 10^12^ genomic particles/ml. The syringe was allowed to remain at the site for 10 min to prevent the backflow of the injected solution. After the surgery and every 24 h for the next 2 days, each rat received 1 μl/g s.c. injection of analgesic carprofen (5 mg/ml). Recovery of the rats was observed closely by the experimenter for 48 h post-surgery.

### Quantitative PCR (qPCR)

At the end of Experiment-1, the rats were anesthetized, brains quickly removed from the skull, and left and right hippocampi were snap-frozen and kept at −80°C until processing. The right (CG01-injected) and left (non-injected) hippocampi were homogenized with QIAzol lysis reagent (Qiagen, Copenhagen, Denmark) and total RNA was extracted using RNeasy Lipid Tissue Kit (Qiagen) following the manufacturer’s protocol. On-column DNAse I treatment was performed using RNase-Free DNase Set (Qiagen). RNA purity (260 nm/280 nm ratio) and concentration of the samples were measured on NanoDrop (Thermo Fischer Scientific) and 0.5 μg of RNA was used for cDNA synthesis following High Capacity cDNA Reverse Transcription Kit protocol (Applied Biosystems). QPCR was run on a LightCycler 480 (Roche) using SYBR Green I Master kit (Roche). The cycling conditions were: 5 min at 95°C followed by 45 cycles of 10 s at 95°C, 15 s at 60°C and 10 s at 72°C; one cycle of 5 s at 95°C, 1 min at 65°C, and then continuous acquisition mode at 97°C for 5 acquisitions per 1°C; one cooling cycle for 10 s with ramp rate 2.0°C/s.

The expression levels of human *NPY* and *Y2* were normalized to the *hypoxanthine-guanine phosphoribosyltransferase* (*HPRT*) expression level, the gene with the least variable expression among several tested reference genes, i.e., *beta-actin* (*ACTB*), *glyceraldehyde-3-phosphate dehydrogenase* (*GAPDH*), *ribosomal protein L13a* (RPLI3A), *tyrosine 3-monooxygenase/tryptophan 5-monooxygenase activation protein zeta polypeptide* (YWHAZ; see Supplementary Figure 1) and thus used as a reference gene in this study. Primers were acquired from Eurofins Genomics and sequences are shown in Table 1. Data are presented as increases in the cycle threshold (Ct) values for *NPY* or *Y2* expression levels in the CG01-injected side minus the non-injected side normalized to *HPRT* and subsequently inverted (multiplied with −1) to visualize increases as positive values.

**Table 1:** Primers used for quantitative PCR (qPCR) experiment.

Gene name	Forward primer sequence (5′-3′)	Reverse primer sequence (5′-3′)
*NPY*	GGAGGACATGGCCAGATACT	ATCTCTGCCTGGTGATGAGG
*Y2*	GGCCATCTTCCGGGAGTATT	GCCAGGCCACTTTTCAGTAC
*ACTB*	TGTCACCAACTGGGACGATA	GGGGTGTTGAAGGTCTCAAA
*GAPDH*	TCACCACCATGGAGAAGGC	GCTAAGCAGTTGGTGGTGCA
*HPRT*	GCAGACTTTGCTTTCCTTGG	CGAGAGGTCCTTTTCACCAG
*RPLI3A*	ACAAGAAAAAGCGGATGGTG	TTCCGGTAATGGATCTTTGC
*YWHAS*	TTGAGCAGAAGACGGAAGGT	GAAGCATTGGGGATCAAGAA

### Cognitive Testing

Only rats from Experiment-2 were subjected to behavioral testing starting on day 21 post-surgery.

#### Y Maze Test

The Y maze, also called spontaneous alternation (SA) test, uses the nature of the rodents to explore the unrestricted areas and their tendency to enter the new area rather than the one previously visited (Momeni et al., 2015). The rats were handled for 2 days before the test and habituated to the test room for 1 h on the test day. The Y maze was composed of three opaque arms distributed 120° apart from each other. The arms were marked A, B, and C, where B was the introductory arm. The maze was surrounded by black curtains with four cues allocated around the maze. On the trial day, each rat was placed in arm B, facing the closed end of the arm, and allowed to freely explore the arms for 12 min. The sequence of the arm entries was recorded. Between testing of each rat, the maze was cleaned with water to reduce olfactory cues. To assess spatial working memory, the percentage of SA was calculated as follows: SA% = [number of alternations/(total number of entries – 2)] × 100. A lower alternation percentage indicates lower spatial working memory (Momeni et al., 2015).

#### Morris Water Maze Test

The Morris water maze was conducted as described previously (Soud et al., 2019). Briefly, a water maze pool (160 cm in diameter, 60 cm high) was filled with 21°C (±1°C) warm water. The pool was virtually divided into four quadrants, and the escape platform (10 cm in diameter) was placed in one of the quadrants, submerged 1.5 cm under the water surface. The area of the water maze was surrounded by black curtains with four visual orientation cues glued on the inner side. Before the test, the rats were handled for 2 days. In the reference memory training, each rat was subjected to three trials for three consecutive days. On each of the trial days, the rat was removed from its home cage and carefully placed on the introductory line in the pool. On each trial the rat was allowed to freely explore the pool until the platform was found, but for a maximum of 90 s. The rats that failed to find the platform during this period were gently guided to the platform. The rats were allowed 20 s orientation time on the platform before being removed from it. Probe tests were performed on 7 and 14 days after the last trial day. During the probe test, the rats performed two trials. During the first trial, the platform was not present in the pool and the rat was allowed to explore the pool for 60 s. Then the platform was gently reintroduced to its usual position in the pool and the rat was guided to the platform where it was allowed to spend 20 s. A second trial was performed like previous trials on training days where the rat was allowed to explore the pool for a maximum of 90 s with the platform in its usual position and allowed to stay on the platform for 20 s.

Using the SMART 3.0 Video Tracking System (Panlab, Harvard, UK) the learning/short-term memory abilities in the test animals were assessed as calculated by the mean latency to reach the platform for each training day. To estimate more long-term spatial memory, the time spent in the platform quadrant on the probe test days was calculated. Swim speed of the animals during the training days was also measured to determine potential effects on motor activity.

### NPY Immunohistochemistry

Only rats from Experiment-2 were used for this analysis. Animals were anesthetized by isoflurane mixture, the brains were quickly removed from the skull, snap-frozen in powdered dry ice, and kept in a −80°C freezer. Rat brains were cut into 14-μm coronal sections on Cryostat CM3050S (Leica), and sections covering the dorsal and ventral hippocampus were collected on SuperFrost PLUS slides and kept on −80°C until use. The slides were defrosted and fixed in 4% paraformaldehyde for 20 min and subsequently washed three times in potassium PBS (KPBS) for 10 min. The sections were then blocked in 10% normal goat serum (NGS), 0.25% Triton X-100 in KPBS for 1 h. Slides were incubated overnight with rabbit anti-NPY antibody (1:500, Sigma–Aldrich, #N9528) diluted in 5% NGS, 0.25% Triton X-100 in KPBS. After washing in KPBS, slides were incubated with secondary goat anti-rabbit Alexa555Plus antibody (1:500, Invitrogen, #A32732), diluted in the same buffer as the primary antibody for 2 h. The slides were washed in T-KPBS for 10 min and two times in KPBS for 10 min. The sections were cover-slipped with the anti-fade mounting medium DABCO (Sigma–Aldrich). Images were acquired on a fluorescence microscope (Olympus BX61 microscope) using the CellSens software. Histological evaluation of the levels of NPY-immunoreactivity was performed using ImageJ 1.49 by densitometric measurements of optical densities in the dentate gyrus, CA3, and CA1 areas of the dorsal and ventral hippocampus by an experimenter blinded to vector treatment of the animals.

For co-staining experiments, sections were additionally blocked in the same blocking solution and incubated with either mouse anti-NeuN (1:100, Merch Millipore; #MAP377) or mouse-anti GFAP (1:500, Sigma–Aldrich; #G3895) overnight followed by Alexa488-conjugated donkey anti-mouse (1:200, Thermo Fisher Scientific; #A21202) or with rabbit-anti Olig2 antibody conjugated with Alexa488 (1:100, Abcam; Ab225099) for 2 h.

### Y2 Functional Binding

Functional binding was performed as previously described (Woldbye et al., 2010). Sections were defrosted and air-dried for 30 min at room temperature (RT), rehydrated in assay buffer A (50 mM Tris-HCl, 3 mM MgCl, 0.2 mM EGTA, 100 mM NaCl, pH 7.4) for 10 min at RT and then preincubated in assay buffer B, composed of assay buffer A supplemented with 0.2 mM dithiothreitol, 1 μM 1,3-dipropyl-8-cyclopentylxanthine (DPCPX, Sigma–Aldrich; #C101), 0.5% w/v BSA, and 2 mM guanosine-5′-diphosphate (GDP; Sigma-Aldrich, DK) for 20 min at RT. Further, the slides were incubated in assay buffer B supplemented with 40 pM [^35^S]-GTPγS (1,250 Ci/mmol; NEG030H250UC; PerkinElmer, DK) for 1 h at 25°C in the presence of NPY peptide (Schafer-N, Copenhagen, DK) at 10^−6^ M to which the Y1 receptor antagonist BIBP3226 (10^−6^ M; Bachem AG, Switzerland; #4034548) and the Y5 receptor antagonist L-152,804 (10^−5^ M; Tocris Cookson, UK; #1382) were added to specifically visualize functional Y2 binding only. To confirm the specificity of this binding assay, the Y2 receptor antagonist BIIE0246 (10^−6^ M; Tocris Cookson, UK; #1700) was added to NPY together with BIBP3226 and L-152,804 at concentrations as above to block Y2 receptor functional binding. Basal binding was determined by incubation in assay buffer B supplemented with 40 pM [^35^S]-GTPγS (1,250 Ci/mmol) without NPY receptor ligands. Since all used NPY receptor antagonists were dissolved in DMSO, DMSO was also added to other incubation buffers (0.1% as final concentration). Incubation was terminated by 2 × 5 min washing in ice-cold 50 mM Tris-HCl buffer (pH 7.4). Sections were dried and exposed to Kodak BioMax MR films together with ^14^C-microscales (Amersham Life Sciences) for 5 days at −20°C. Films were developed in Kodak GBX developer. Y2 receptor functional binding levels were measured in the dorsal/ventral hippocampus by an experimenter blinded to vector treatment of the animals as previously described (Christensen et al., 2006).

### Statistical Analysis

Statistical analysis was performed with GraphPad Prism v8.4.3. Non-parametric Kruskal–Wallis ANOVA test followed by Wilcoxon matched-pairs signed rank test was used for immunohistochemistry and functional binding data while parametric one-way ANOVA or two-way repeated measures mixed model ANOVA were used for body weight and behavioral data. Potential correlation between CG01-mediated overexpression of NPY/Y2 (ratios of ipsilateral vs. contralateral sides) and behavioral performance was analyzed using Spearman’s correlation. *P* < 0.05 was considered significant.

## Results

### No Effect of the Vector Treatment on Body Weight

**Figure 2:**
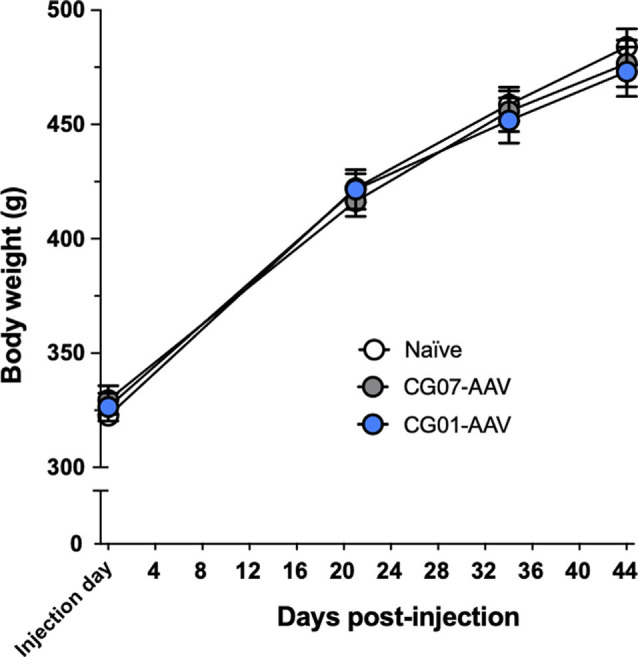
Body weight did not differ between the three groups during Experiment-2 (*n* = 12 per group).

No signs of suffering or discomfort was observed in the animals before and after the vector injections during the whole course of the experiments. NPY is a known orexigenic agent in the hypothalamus, causing prominent increase in food intake and body weight (Loh et al., 2015) and, consequently, the animals were observed for potential weight gain (Figure 2). No significant differences were found between the three groups with regard to body weight (non-significant treatment effect in repeated measures two-way ANOVA: *F*_(2,33)_ = 0.073, *P* = 0.929).

### Long-Term Expression of NPY and Y2 Transgenes

**Figure 3:**
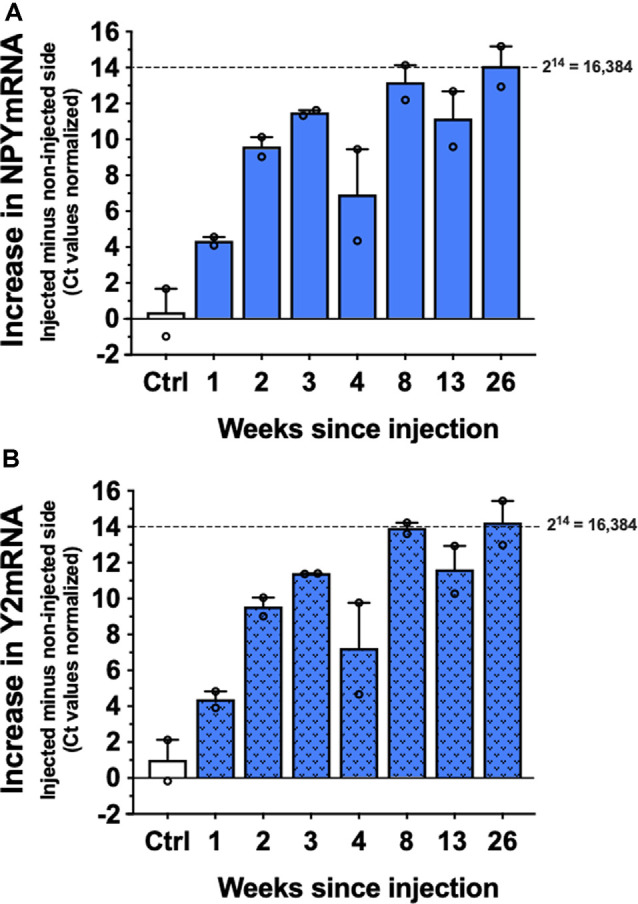
Levels of NPYmRNA **(A)** and Y2mRNA **(B)** at different time points after right intrahippocampal CG01 injection or in non-injected controls (Ctrl) normalized to Hypoxanthine-Guanine Phosphoribosyltransferase (HPRT) levels (*n* = 2). The graphs show the increases in normalized Ct values in the injected side subtracted the non-injected side. Columns are means with SEM values as error bars. Individual values are depicted as small circles.

As evident from Figure 3, the expression of both CG01 vector-encoding genes, NPY and Y2, were upregulated at week 1 and appeared to reach close to maximum after 3 weeks. Both transgene expressions showed no sign of decay as long as 26 weeks after CG01.

### CG01-Mediated Overexpression of NPY and Y2 as Revealed by NPY Immunohistochemistry and Functional Y2 Binding

**Figure 4:**
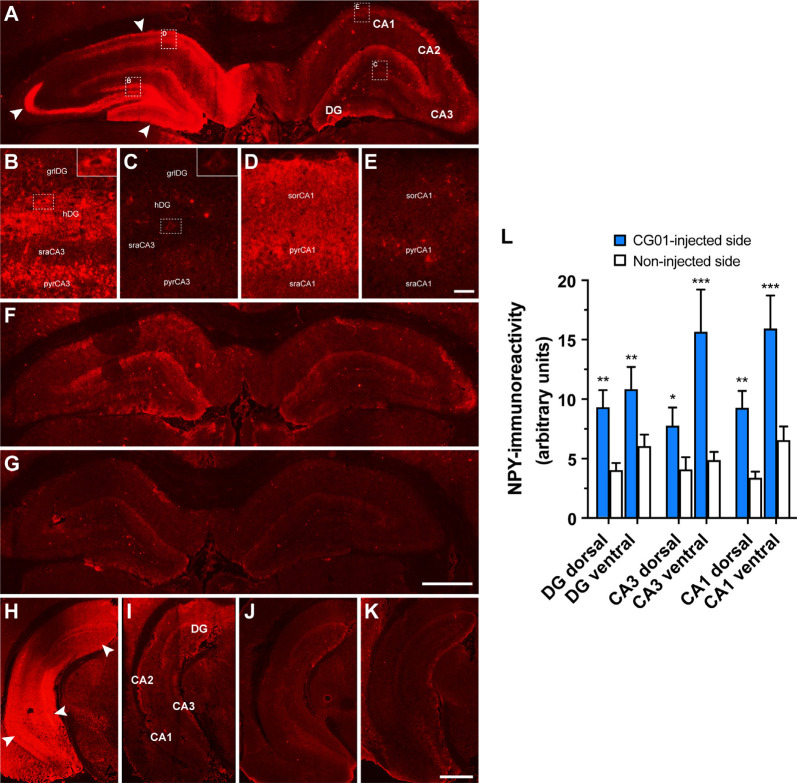
Increased neuropeptide Y (NPY)-immunoreactivity shown in the right dorsal hippocampal dentate gyrus (DG), CA3, and CA1 of a CG01-injected rat (white arrows) compared to the contralateral non-injected side **(A)**. High-magnification images of hatched areas in panel **(A)**, showing increased NPY-immunoreactivity in the granular layer (grlDG) and hilus (hDG) of the DG and adjoining CA3 stratum radiatum (sraCA3) and pyramidal layer (pyrCA3) ipsilaterally **(B)** as compared to contralaterally **(C)** to the injection, as well as ipsilateral CA1 stratum oriens (sorCA1), pyramidal layer (pyrCA1), and stratum radiatum (sraCA1) **(D)** as compared to contralateral non-injected side **(E)**. Inserts in right top corners of **(B)** and **(C)** show increased NPY-immunoreactivity in dentate hilar interneurons in ipsilateral compared to contralateral side. NPY-immunoreactivity shown in a CG07-injected rat **(F)** and in a naïve rat **(G)**. Similarly, increased NPY-immunoreactivity shown in the DG, CA3, and CA1 in the ventral part of the hippocampus of a CG01-injected rat (white arrows; **H**) compared to non-injected hippocampus of the same rat **(I)**. NPY-immunoreactivity in the ventral hippocampus of CG07-injected rat **(J)** and in naïve rat **(K)**. Magnification bars = 1 mm in panels **(A,F,G)**, 50 μm in panels **(B–E)**, and 1.5 mm in panels **(H–K)**. Densitometric measurements confirmed that CG01 increased NPY-immunoreactivity in dorsal and ventral parts of hippocampus compared to the contralateral non-injected side **(L)**. *P < 0.05, **P < 0.01, ***P < 0.001 vs. non-injected side, Kruskal–Wallis ANOVA followed by Wilcoxon matched-pairs signed rank tests. Data are means ± SEM (*n* = 10–12).

To confirm proper CG01-mediated overexpression of NPY and Y2 transgenes, the brains of the rats undergoing memory testing were processed for NPY immunohistochemistry and Y2 functional binding. As expected, NPY immunohistochemical examination of the rat brains revealed increased NPY-immunoreactivity ipsilateral to the CG01-injection in the hippocampal regions dentate gyrus, CA3, and CA1 both at dorsal and ventral levels compared to the contralateral non-injected side (Figures 4A,H,I) as confirmed with densitometric measurements (Figure 4L). In contrast, NPY-immunoreactivity was modest and without side differences in CG07-injected and naïve rats (Figures 4F,G,J,K). High-magnification images (Figures 4B–E) showed that increased NPY-immunoreactivity in the DG was mainly observed in mossy fibers and interneurons in the hilus (inserts of Figures 4B,C), but some labeling was also seen in cell bodies in the granular layer (Figures 4B,C) and in the molecular layer (Figure 4A). In the dorsal CA3, increased NPY-immunoreactivity was particularly strong in the stratum lucidum (Figure 4A), but labeling was also observed in some cell bodies of the CA3 pyramidal layer, particularly in the CA3c region (Figures 4B,C). At ventral levels of CA3, labeling was higher (Figure 4L), also in the pyramidal layer of ventral CA3 (Figures 4H,I). In the dorsal CA1, elevated NPY-immunoreactivity was observed in the pyramidal layer (Figures 4D,E) and stratum oriens, but less so in the stratum radiatum where CA3 pyramidal projections terminate. As for the hippocampal CA2, more than two thirds of the CG01-injected animals were also observed to display increased NPY-immunoreactivity ipsilaterally vs. contralaterally in the dorsal (not shown) and ventral (Figures 4H,I) parts.

**Figure 5:**
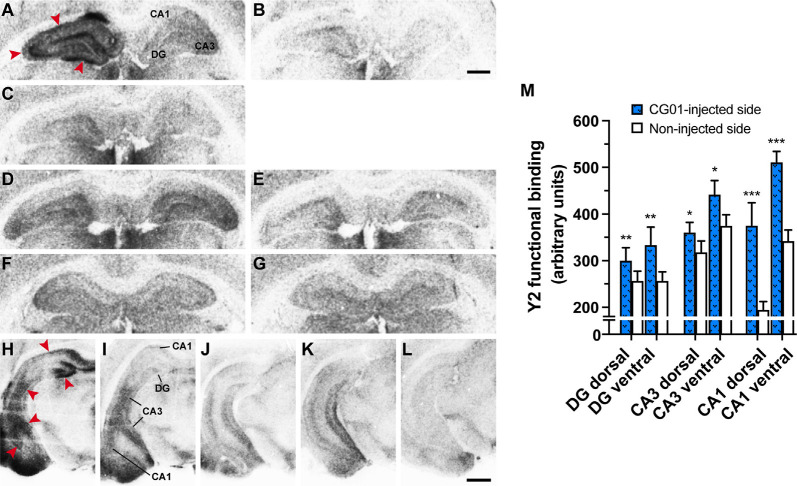
Unilateral intrahippocampal administration of CG01 increased Y2 transgene expression as seen by increased Y2 functional binding (NPY + Y1 antagonist + Y5 antagonist) in the right dorsal hippocampal DG, CA3, and CA1 of a CG01-injected rat (red arrows) compared to the contralateral non-injected side **(A)**. Basal binding (no addition of NPY; **B**) and blocking of Y2 binding (NPY + Y1 antagonist + Y5 antagonist + Y2 antagonist; **C**) are shown in CG01-injected rat. Y2 functional binding is shown in CG07-injected **(D)** and in naïve rat **(F)** with corresponding basal binding (**E** and **G**, respectively). Increased Y2 functional binding was also seen in DG, CA3, and CA1 in ventral part of the CG01-injected hippocampus (red arrows; **H**) compared to non-injected hippocampus **(I)**. Y2 functional binding displayed in ventral hippocampus of CG07-injected rat **(J)** and in naïve rat **(K)** while **(L)** shows basal binding in CG01-injected ventral hippocampus. Magnification bars = 1 mm in panels **(A–G)** and 1.5 mm in panels **(H–L)**. Densitometric measurements confirmed that CG01 increased Y2 functional binding in dorsal and ventral parts of hippocampus after unilateral intrahippocampal administration compared to the contralateral non-injected side **(M)**. *P < 0.05, **P < 0.01, ***P < 0.001 vs. non-injected side, Kruskal–Wallis ANOVA followed by Wilcoxon matched-pairs signed rank tests. Data are means ± SEM (*n* = 10–12).

As evidence that the [^35^S]-GTPγS Y2 functional binding assay was working, there was a clear increase in Y2-stimulated (NPY + Y1 antagonist + Y5 antagonist) binding (Figures 5A,D,F,H,J,K) compared to basal binding (Figures 5B,E,G,L) in all treatment groups. Consistent with the NPY-immunoreactivity results, Y2 functional binding was also increased in CG01-injected DG, CA3, and CA1 regions ipsilaterally compared to the noninjected side both at dorsal and ventral levels (Figures 5A,H,I), and as confirmed by densitometric measurements (Figure 5M). As confirmation that increased labeling was due to Y2 functional binding, the signal was blocked after addition of Y2 antagonist (Figure 5C). Thus the assays confirmed that both NPY and Y2 transgenes were well expressed in all the studied hippocampal regions on the injected side. In general, NPY-immunoreactivity and/or functional Y2 binding were confined to the DG and hippocampus proper, but in some animals, particularly at ventral levels, some vector-mediated expression was also observed to spread into the subiculum (Figure 5A) and adjoining areas, including the entorhinal cortex, amygdalopiriform transition area, and cortical amygdaloid nuclei (Figures 4H,I, 5H,I).

### NPY Expression in Hippocampal Cells (Tropism)

**Figure 6:**
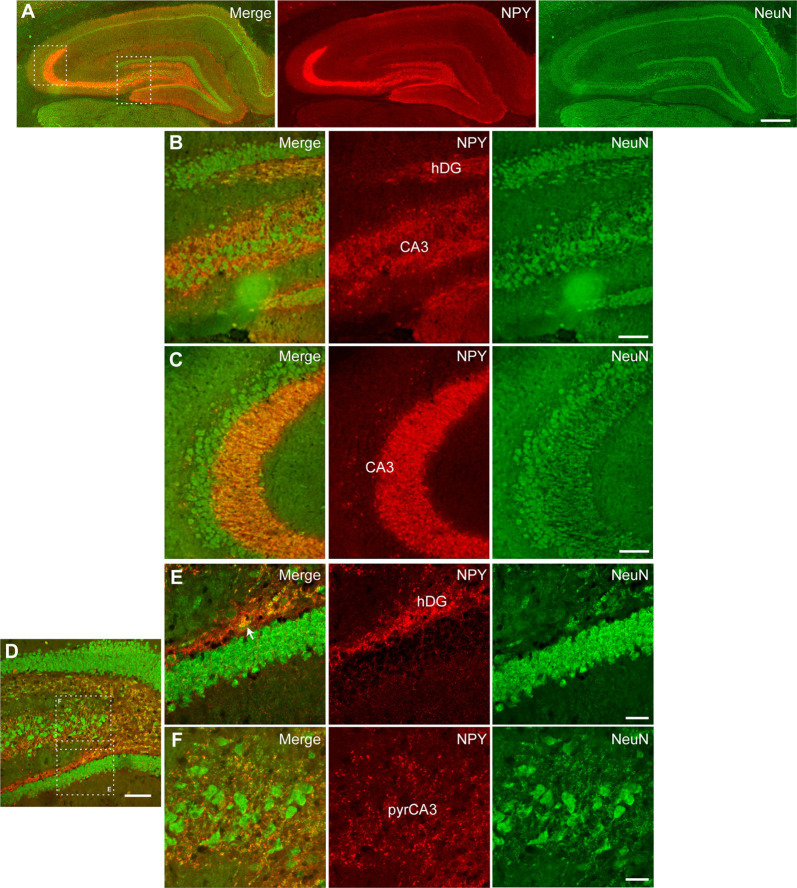
Neuronal tropism in the dorsal hippocampal DG and CA3 of CG01-treated rat as evidenced by NPY-immunoreactivity being co-expressed with NeuN-immunoreactivity as revealed by fluorescent **(A–C)** and confocal microscopy **(D–F)**. **(A)** Fluorescent microscopy overview images of NPY/NeuN co-staining (merge), NPY- and NeuN-immunoreactivity, with high-magnification images of hatched areas in panel **(A)**, showing hilus of the DG (hDG; **B**) and CA3 **(B,C)**. **(D)** Confocal microscopy overview image of NPY/NeuN co-staining, with high-magnification images of hatched areas in panel **(D)**, showing co-labeling in fibers and interneuron (white arrow) in hDG **(E)** and fibers in pyramidal layer of CA3 (pyrCA3; **F**). Scale bars: 500 μm **(A)**, 100 μm **(B–D)**, and 30 μm **(E,F)**.

**Figure 7:**
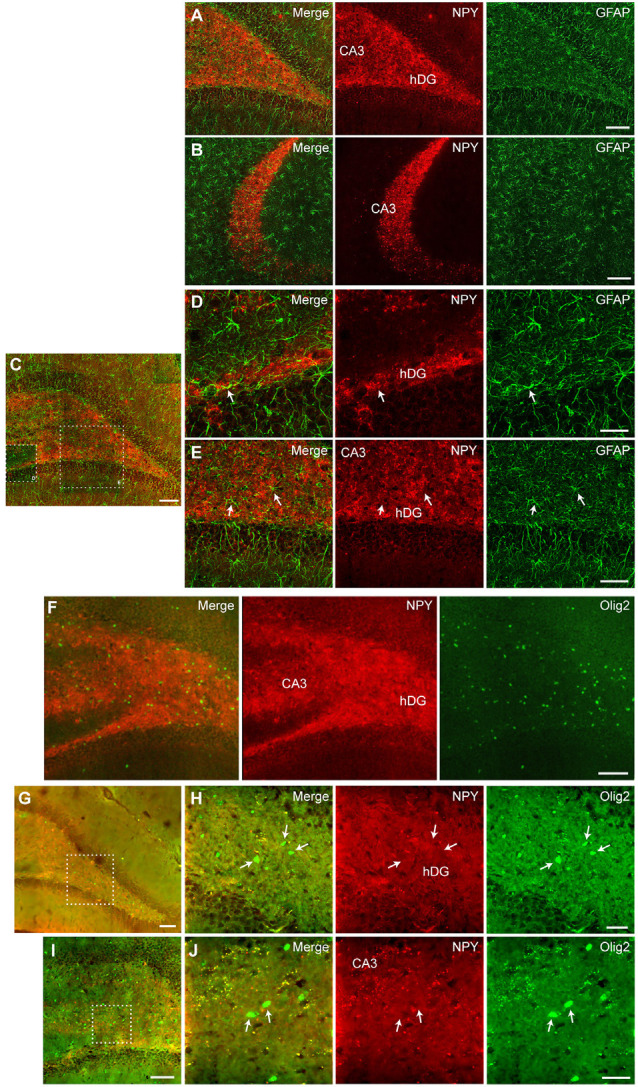
Fluorescent **(A,B,F)** and confocal microscopy **(C–E,G–J)** revealed no co-staining between NPY and GFAP **(A–E)** or Olig2 **(F–J)** in the dorsal hippocampus after CG01 treatment, suggesting that CG01 does not express in astrocytes or oligodendrocytes. Fluorescent microscopy images of NPY/GFAP co-staining (merge), NPY- and GFAP-immunoreactivity in the dentate hilus (hDG; **A**) and CA3 **(A,B)** showing no overlap between NPY- and GFAP-immunoreactivity. **(C)** Similarly, confocal microscopy overview image shows no overlap between NPY- and GFAP-immunoreactivity in the DG and CA3, with high-magnification images of hatched areas in panel **(C)**, showing hDG **(D)** and CA3 (**D,E**; white arrows indicate astrocytes). **(F)** Fluorescent microscopy overview images of NPY/Olig2 co-staining (merge), NPY- and Olig2-immunoreactivity showing no overlap in the hDG and CA3 of the dorsal hippocampus. **(G,I)** Confocal microscopy overview images of NPY/Olig2 co-staining, with high-magnification images of hatched areas in panels (**G** and **I**), showing no overlap in the dentate hilus **(H)** and CA3 (**J**; white arrows indicate oligodendrocytes). Scale bars: 100 μm **(A–C,F,G,I)**, 30 μm **(D,H,J)**, and 60 μm **(E)**.

Further, we performed immunohistochemical co-staining to investigate the cell populations expressing NPY after CG01-treatment in the dorsal dentate gyrus and CA3. Co-staining for NPY and the neuronal marker NeuN showed extensive overlap of immunoreactivity in neuronal fibers particularly from dentate granule neurons passing through the dentate hilus to terminate in the CA3 stratum lucidum (Figures 6A–F) and interneurons in the dentate hilus (Figure 6E) while cells expressing GFAP- or Olig2-immunoreactivity did not appear to co-express NPY (Figures 7A–J). This suggests that CG01-mediated hippocampal NPY overexpression predominantly targets neurons.

### No Effects of CG01 On Short- or Long-Term Memory

**Figure 8:**
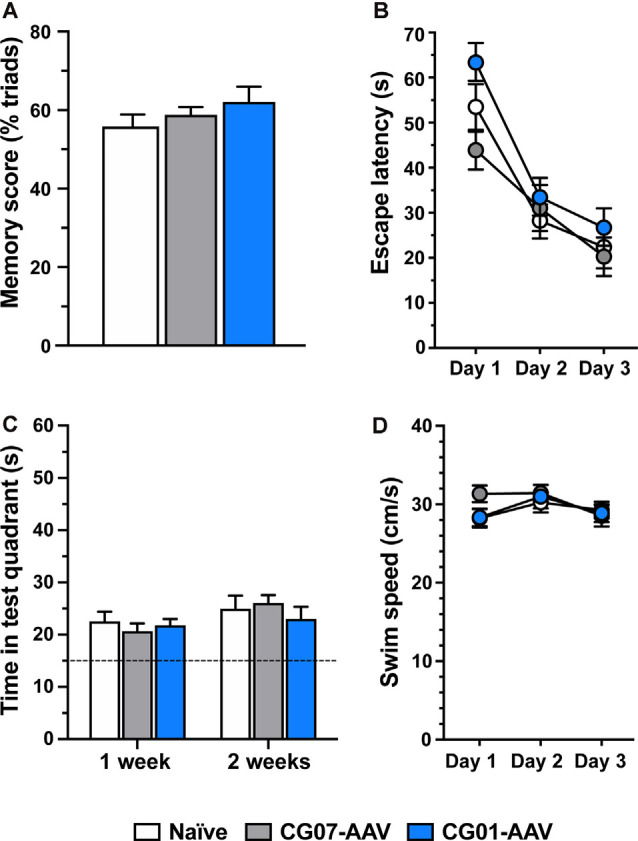
CG01 unilateral intrahippocampal administration did not significantly affect learning and memory function (% triads) compared to CG07-AAV control vector treatment or compared to treatment naïve control rats as revealed by testing in the Y-maze test (**A**; *n* = 11–12 rats per group). Similar result was obtained in the Morris water maze test where escape latencies during learning sessions **(B)** and during probe tests 1 and 2 weeks after **(C)** did not differ significantly between the groups (*n* = 11–12 rats per group). Likewise, swim speed did not differ between the groups during the learning trials **(D)**.

In order to check whether the injection of CG01 could affect cognitive function of the animals, two memory tests were carried out. Using the Y-maze SA test, which is a simple test evaluating spatial memory (Gøtzsche and Woldbye, 2016), no significant effect was detected between CG01 vector treated compared to the control groups, as revealed by percentage triads conducted during the test (Figure 8A; one-way ANOVA: *F*_(2,32)_ = 0.98, *P* = 0.39). Similarly, using the Morris water maze test, which is a more complex test used to evaluate learning as well as short- and long-term memory (Morris et al., 1986; Vorhees and Williams, 2006), no overall significant effect of CG01 was revealed during the three training days (Figure 8B; non-significant treatment effect in repeated measures two-way mixed effects model: *F*_(2,33)_ = 3.24, *P* = 0.052) nor during the probe tests (Figure 8C; *F*_(2,65)_ = 0.28, *P* = 0.76). However, during the training days, there was a clear effect of time (*F*_(1.96,63.7)_ = 42.69, *P* < 0.0001) and no evidence of interaction (*F*_(4,65)_ = 1.21, *P* = 0.32), indicating respectively that all groups learned the memory task of finding the escape platform and did this equally well. As confirmation that the animals of all groups remembered the location of the platform, all groups displayed a mean time spent in the test quadrant clearly above the theoretical 25%, i.e., 15 s out of the total 60 s probe test periods (Figure 8C, dashed line). Swim speed during the learning sessions also did not differ significantly between the groups (Figure 8D; *F*_(2,33)_ = 0.54, *P* = 0.59), indicating that treatment with the CG01 vector also had no locomotor side effects that could have influenced the memory responses measured in this test. Performance in the memory tests after CG01-treatment was not correlated in any of the measured hippocampal regions to levels of NPY-immunoreactivity (Spearman *r* performed on ratios of ipsilateral vs. contralateral non-injected sides: Y-maze: *P* = 0.09–0.81; Morris water maze, days 1–3, probe tests: *P* = 0.11–0.96) or Y2 functional binding (Spearman *r*: Y-maze: *P* = 0.22–0.86; Morris water maze, days 1–3, probe tests: *P* = 0.12–0.97).

## Discussion

Currently, gene therapy is being developed for pharmacoresistant temporal lobe epilepsy using unilateral intrahippocampal gene therapy with CG01, an AAV vector mediating overexpression of NPY and Y2 (Drew, 2018). Our groups have explored the effects of unilateral NPY/Y2 gene therapy and shown that hippocampal overexpression of both transgenes induces significant seizure-suppressant effect in a chronic rat temporal lobe epilepsy model (Ledri et al., 2016; Melin et al., 2019). Potential side effects should be fewer or less pronounced after unilateral hippocampal gene therapy. Consistent with this view, unilateral surgical excision of a large part of hippocampus and amygdala of the epileptic focus is considered acceptable with regards to cognitive side effects (Sheikh et al., 2019). Thus to pave the way for future clinical testing, the present study provides important knowledge of the effects of CG01 in adult rat hippocampus on the duration of transgene expression, cellular tropism, and potential side effects on body weight and cognitive function in rats.

As for the duration of CG01-mediated transgene overexpression, the present study shows that there are prominent increases in both NPY and Y2 mRNA levels in the injected hippocampus compared to the non-injected side, reaching close to maximum after 3 weeks and remaining at this level or at even higher levels as long as 26 weeks after CG01 vector injection. Twenty-six weeks may correspond to around 13–18 years of lifetime in humans (Andreollo et al., 2012; Agoston, 2017) and suggests that CG01-mediated expression of NPY and Y2 transgenes could last for long duration in humans. Consistent with this view, AAV vector-mediated overexpression has been detected as long as 10 years post-surgery in Parkinson’s disease patients (Chu et al., 2020) and remained unchanged for 15 years in non-human primates (Sehara et al., 2017).

Having established that NPY-immunoreactivity was clearly upregulated by CG01 treatment, the cellular distribution of transgene expression (i.e., tropism of the AAV viral vector) was explored using immunohistochemical co-staining between NPY and specific cell markers in hippocampal dorsal dentate gyrus and CA3. NPY-immunoreactivity was found to co-localize in NeuN-positive neuronal fibers and cells in the dentate hilus and mostly fibers in the CA3 region. In contrast, no co-staining was seen between NPY- and GFAP- or Olig2-immunoreactivity, indicating that, in the hippocampus, CG01 predominantly, if not exclusively, mediates transgene expression in neurons and not in astrocytes or oligodendrocytes. Cellular tropism is driven by the serotype and promotor (Watakabe et al., 2015; Hudry and Vandenberghe, 2019). *In vitro*, the AAV1 serotype has been shown to transduce preferentially pyramidal neurons in CA3/CA1 of primary hippocampal cultures, but also to a minor degree astrocytes when using the hCMV promoter (Royo et al., 2008). Nonetheless, consistent with the present study, *in vivo*, the AAV1 vector transduced neurons in the dentate granular layer and pyramidal neurons of the CA3/CA1 (Burger et al., 2004), and another study using AAV1 to induce hippocampal NPY expression also reported predominantly neuronal expression (Noe et al., 2010). Further consistent with the present findings with CG01, when targeting the hippocampus of adult rats with an AAV1 vector utilizing the CAG promoter, selective transduction of neurons was observed without transduction of astrocytes or microglia (Jeon et al., 2015). Previous data suggest a minor transduction of oligodendrocytes when injecting an AAV1 into mice (Wang et al., 2003), but in contrast to these findings we did not observe expression of NPY in oligodendrocytes in rats.

NPY has powerful feeding stimulatory effects by acting in the hypothalamus (Loh et al., 2015). Since AAV-mediated bilateral overexpression of NPY in limbic rodent brain regions, including the hypothalamus (Tiesjema et al., 2007) and amygdala (Christiansen et al., 2014), has been associated with increased body weight, we also measured body weight in the present study. No significant effects on body weight were observed after unilateral hippocampal CG01 administration compared to both CG07 control vector administration and naïve rats. This is consistent with several previous studies indicating that targeting hippocampus with vectors mediating overexpression of NPY is not associated with weight gain (Richichi et al., 2004; Woldbye et al., 2010; Christiansen et al., 2014; Soud et al., 2019).

Central administration of NPY is known to have both inhibitory and stimulatory effects on memory, depending on the brain region, dose, memory test, and application time point in the learning process (for review see Gøtzsche and Woldbye, 2016). Experiments with Y2 receptor knockout mice and Y2 receptor antagonist indicate that Y2 receptors play an important role in mediating hippocampal memory-related effects of NPY (Redrobe et al., 2004; Gonçalves et al., 2012; Hörmer et al., 2018). Studies with AAV vectors encoding NPY have shown that hippocampal NPY overexpression may attenuate the memory-related synaptic phenomenon long-term potentiation (LTP) *in vitro* (Sørensen et al., 2008a,b). Similarly, direct NPY application inhibits hippocampal LTP (Whittaker et al., 1999; Sørensen et al., 2008b). However, in electrically kindled animals (a chronic epileptic condition), LTP was not further decreased after NPY-AAV treatment compared to control vector (Sørensen et al., 2009). This suggests that although naïve rodents may display reduced LTP after NPY-AAV treatment, this is not evident in epileptic animals. This is consistent with the finding in TLE patients that they may experience memory impairment (Elger et al., 2004), and suggests that future treatment with NPY gene therapy may not additionally impair their memory. Nonetheless, *in vivo*, bilateral AAV-mediated hippocampal NPY overexpression has been shown to transiently inhibit memory and learning in a two-platform spatial discrimination water maze test in rats (Sørensen et al., 2008a). Thus, on days 3 and 4 of training, the rats appeared to remember the location of the correct platform less well than control vector-treated rats, but on the last days of training (i.e., days 5–7), NPY-AAV treated rats were equally good as controls. In two other studies, no significant effect was found after bilateral NPY-AAV treatment in seizure-naïve rats in the same model (Noè et al., 2008; Noe et al., 2010). The reason for this discrepancy is not clear, it may be relevant that the latter studies used a CAG promotor (as opposed to neuron-specific enolase promotor; Sørensen et al., 2008a), serotype 1 (as opposed to mixed serotype 1/2) and injected the vector in both the septal temporal parts of hippocampus (as opposed to only septal). Consistent with the lack of effect on hippocampal mediated memory, NPY-AAV treatment also did not influence memory in a passive avoidance test (Noe et al., 2010). Similarly, our group recently showed that bilateral septal/temporal hippocampal overexpression of NPY under a synapsin promotor and with mixed serotype 2/8 also did not influence memory in a Morris water maze test (Soud et al., 2019).

No previous studies have examined potential effects on memory after vector-mediated Y2 overexpression in normal rodents. However, re-expression of Y2 receptors in the dorsal hippocampus of Y2 knockout mice decreased spatial memory in mice (Hörmer et al., 2018). The present study using an AAV vector (CG01) with a CAG promotor to induce overexpression of both Y2 and NPY in septal and temporal hippocampus was not associated with a significant effect on short-term or long-term memory using the hippocampus-dependent Morris water maze test. Consistent with this finding, the Y maze SA test also showed no significant difference in task performance between CG01 and the two control groups, indicating no differences in working memory. Finally, no effect was seen on locomotor activity in the water maze, suggesting that locomotor effects were also not influenced by CG01 treatment. Noe et al. (2010) also did not find significant effects on locomotion after NPY-AAV treatment. Taken together, these data indicate that CG01-mediated overexpression of NPY and Y2 does not significantly influence memory in the tested animal models.

In conclusion, the present study showed that unilateral CG01-mediated overexpression of NPY and Y2 transgenes in the dorsal and ventral parts of the hippocampus was not associated with significant effects on learning and memory as revealed by the Y-maze and Morris water maze memory tests in adult male rats. No effects of CG01 administration were seen on body weight either. CG01-mediated levels of NPY/Y2 transgene expression were long-lasting, remaining at maximum levels all the way to the last time point of the study, 26 weeks, after intrahippocampal injection. CG01 appeared to selectively induce transgene expression in hippocampal neurons. These data suggest that treatment with CG01 in future clinical trials for pharmacoresistant temporal lobe epilepsy patients should not have significant side effects on body weight or memory.

## Data Availability Statement

The raw data supporting the conclusions of this article will be made available by the authors, without undue reservation.

## Ethics Statement

The animal study was reviewed and approved by Danish Animal Experiments Inspectorate.

## Author Contributions

JS, KD, EM, SR, and DW conducted the experimental work and analyzed the data. AE, KA, EM, MK, and DW conceived and designed the study. JS, SP, KD, MK, and DW wrote the manuscript. All authors contributed to the article and approved the submitted version.

## Conflict of Interest

The authors declare that this study received funding in part by CombiGene AB. The funder had no role in data collection, analysis, or decision to submit for publication, however, the funder participated in the study design and writing of this article. AE and KA are employees at CombiGene, and DW and MK are co-founders and consultants of this company. The remaining authors declare that the research was conducted in the absence of any commercial or financial relationships that could be construed as a potential conflict of interest.

## Supplementary Material

SUPPLEMENTARY FIGURE 1 Different reference genes were tested and HPRT was found to be the least regulated and was consequently used for determining NPY and Y2 mRNA levels. Data are pooled averaged values for both sides of the hippocampus (L + R) at different time points after CG01 injection (*n* = 1–2 rats).
