# Supplementary figures and images for: Gene Therapy Vector Encoding Neuropeptide Y and Its Receptor Y2 for Future Treatment of Epilepsy: Preclinical Data in Rats
Source: Front Mol Neurosci. 2020 Dec 4;13:232. doi: 10.3389/fnmol.2020.603409 (PMC7746806; doi:10.3389/fnmol.2020.603409)

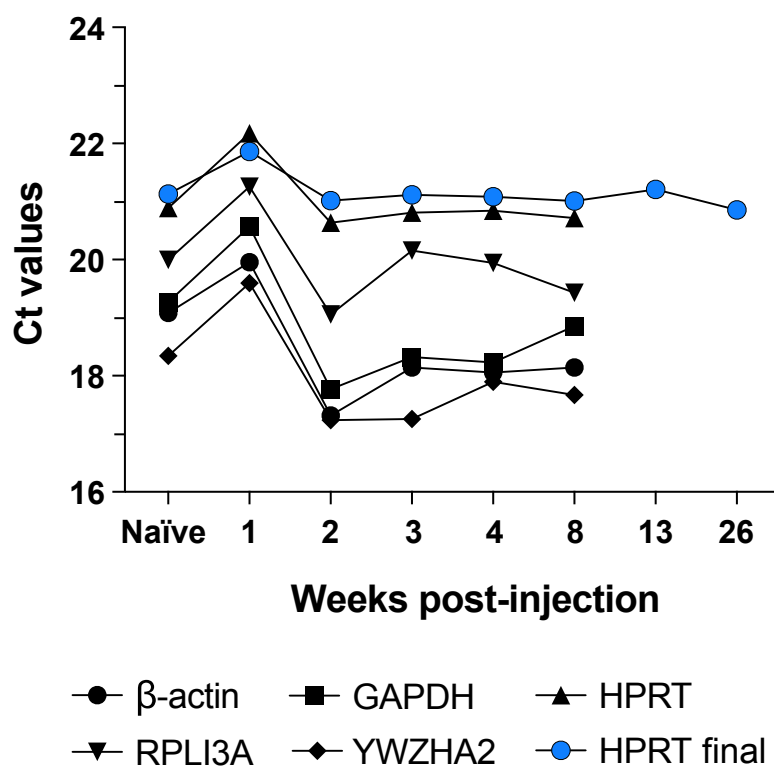

Supplement: SUPPLEMENTARY FIGURE 1 — Different reference genes were tested and HPRT was found to be the least regulated and was consequently used for determining NPY and Y2 mRNA levels. Data are pooled averaged values for both sides of the hippocampus (L + R) at different time points after CG01 injection (n = 1–2 rats). [file Data_Sheet_1.PDF]
